# A genome-wide analysis of *Escherichia coli* responses to fosfomycin using TraDIS-*Xpress* reveals novel roles for phosphonate degradation and phosphate transport systems

**DOI:** 10.1093/jac/dkaa296

**Published:** 2020-08-05

**Authors:** A Keith Turner, Muhammad Yasir, Sarah Bastkowski, Andrea Telatin, Andrew J Page, Ian G Charles, Mark A Webber

**Affiliations:** d1 Quadram Institute, Norwich Research Park, Colney Lane, Norwich NR4 7UQ, UK; d2 Norwich Medical School, Norwich Research Park, Colney Lane, Norwich NR4 7TJ, UK

## Abstract

**Background:**

Fosfomycin is an antibiotic that has seen a revival in use due to its unique mechanism of action and efficacy against isolates resistant to many other antibiotics. In *Escherichia coli*, fosfomycin often selects for loss-of-function mutations within the genes encoding the sugar importers, GlpT and UhpT. There has, however, not been a genome-wide analysis of the basis for fosfomycin susceptibility reported to date.

**Methods:**

Here we used TraDIS-*Xpress*, a high-density transposon mutagenesis approach, to assay the role of all genes in *E. coli* involved in fosfomycin susceptibility.

**Results:**

The data confirmed known fosfomycin susceptibility mechanisms and identified new ones. The assay was able to identify domains within proteins of importance and revealed essential genes with roles in fosfomycin susceptibility based on expression changes. Novel mechanisms of fosfomycin susceptibility that were identified included those involved in glucose metabolism and phosphonate catabolism (*phnC-M*), and the phosphate importer, PstSACB. The impact of these genes on fosfomycin susceptibility was validated by measuring the susceptibility of defined inactivation mutants.

**Conclusions:**

This work reveals a wider set of genes that contribute to fosfomycin susceptibility, including core sugar metabolism genes and two systems involved in phosphate uptake and metabolism previously unrecognized as having a role in fosfomycin susceptibility.

## Introduction

The increasing prevalence of bacteria that are resistant to clinically important antibiotics has led to searches for alternative options to treat problematic infections.^1^ There has been limited progress in the development of new antibiotics and one strategy has been to revive older drugs that may be effective but are not commonly used in clinical practice.^2^ One example is fosfomycin, which has seen an increase in clinical use in recent years. Fosfomycin has a unique mode of action where it targets the initial stages of peptidoglycan biosynthesis by acting as a phosphoenolpyruvate analogue and inhibiting MurA.^3^ Consequently, fosfomycin retains activity against bacterial strains expressing β-lactamases that inhibit later peptidoglycan biosynthetic reactions. This is useful given the prevalence of different β-lactamase enzymes in important pathogens. Fosfomycin is a phosphonic acid molecule produced in nature by *Streptomyces* species and is commonly used for treatment of complicated urinary tract infections and increasingly for more serious systemic infections. In Enterobacteriaceae, fosfomycin enters the cell by acting as a mimic for two nutrient importer systems, GlpT and UhpT.^4^

Resistance to fosfomycin has been shown to be relatively easy to select *in vitro* and resistant mutants often show loss of function of GlpT or UhpT, or have mutations in adenylcyclase (*cyaA*) or phosphotransferase (*ptsI*) genes, both of which control intracellular levels of cyclic AMP, which in turn regulates expression of *glpT* and *uhpT.*^5–9^ In addition, alterations within the drug target MurA (particularly those altering a Cys115 residue near the active site) can decrease susceptibility by reducing its affinity for fosfomycin.^10–12^ Over-expression of *murA* has also been observed in fosfomycin-resistant isolates and is thought to act by saturating the drug.^13^^,^^14^ Resistance to fosfomycin may also be acquired by genetic transfer of *fosA* and *fosB* coding for functions that disrupt the fosfomycin oxirane ring.^15–17^

Whilst it has been easy to select for fosfomycin-resistant isolates *in vitro*, there is evidence that selection of resistance carries a major fitness cost, and that fosfomycin-resistant isolates may be compromised in virulence.^18–20^ Various studies have looked for the prevalence of fosfomycin resistance in different settings, and resistance rates in general have remained relatively low, even in high-use settings.^21^^,^^22^

Given the recent increase in the use of fosfomycin, we used a genome-wide transposon mutagenesis approach in *Escherichia coli* to identify loci involved in fosfomycin susceptibility.^23^ We identified new loci as being involved in fosfomycin susceptibility, including the phosphonate uptake and utilization system and phosphate import system, as well as identifying sub-domains within some proteins involved in fosfomycin susceptibility.

## Materials and methods

### TraDIS-Xpress library

We recently described the construction of a high-density (insert approximately every 6 bp) TraDIS-Xpress library in *E. coli* BW25113, which was used in this work.^24^ BW25113 is a commonly used reference strain, a full and well-annotated genome sequence is available, and it was the parent strain for the KEIO collection of defined insertion mutants, thus enabling easier validation of the roles of candidate genes.^24^ The transposon used {a mini-Tn*5* transposon coding for kanamycin resistance [*aph(3′)-Ia*]} incorporates an outward-transcribing *tac* promoter 3′ of the kanamycin cassette that is inducible by IPTG, allowing over-expression or repression of genes adjacent to insertion sites (depending on insert orientation) as well as gene inactivation. This allows the roles of essential genes in response to a stress to be analysed based on expression changes; traditionally these loci have been cryptic in TraDIS experiments as insertions within them are lethal.

### Fosfomycin exposure conditions and TraDIS-Xpress sequencing

The MIC of fosfomycin for BW25113 was determined using microbroth dilution in Mueller–Hinton (MH) and LB broth (the same growth medium that was used for TraDIS-*Xpress* experiments). For TraDIS-*Xpress* experiments, approximately 10^7^ mutants were inoculated into 1.7 mL LB broth in deep 96-well plates containing doubling concentrations of fosfomycin ranging from 0.25× to 2× MIC (and a drug-free control). Replicate experiments were completed with no induction, or with the addition of 0.2 or 1 mM IPTG to induce transcription from the outward-transcribing promoter. Mutants were grown for 24 h at 37°C. All experiments were performed in duplicate to give a total of 30 independent TraDIS-*Xpress* experiments (Figure S1, available as Supplementary data at *JAC* Online).

After growth under experimental conditions, DNA was extracted from pools of mutants using a Quick-DNA™ Fungal/Bacterial 96 Kit (Zymo Research). DNA was then fragmented using a Nextera DNA library preparation kit (Illumina) except that Tnp-i5 oligonucleotides were used instead of i5 index primers, and 28 PCR cycles. The resulting DNA was sequenced on a NEXTSeq 500 sequencing machine using a NextSeq 500/550 High Output v2 kit (75 cycles). All sequence data have been deposited with EBI under project accession number PRJEB29311.

### Bioinformatics

Results were analysed using BioTraDIS (version 1.4.1) and AlbaTraDIS (version 0.0.5), developed for TraDIS-*Xpress* analysis and recently described.^25^^,^^26^ Briefly, BioTraDIS was used to map sequence reads against the BW25113 reference genome (CP009273) using SMALT and to create insertion plots.

The patterns of inserts were compared between fosfomycin-exposed and control conditions, AlbaTraDIS then calculated the number of inserts within each gene as well as assessing the number of forward and reverse insertions per gene and within a window of 198 bp upstream and downstream of each gene. This length for a window was chosen as likely to include regions within which inserts were likely to influence expression of the relevant gene. The number of sequence reads was modelled on a per-gene basis using a negative binomial distribution and an adapted exact test as implemented in edgeR followed by multiple testing correction to identify significant differences between conditions (each test condition compared with insert patterns from drug-free controls).^27^^,^^28^ A set of default cut-offs for significance and number of reads were applied (*q*-value ≤0.05, logFC ≥1, logCPM >8). This resulted in a list of candidate genes involved in fosfomycin susceptibility as well as a prediction as to whether a change in expression of a gene (either down- or up-regulated) influences survival. The insertion patterns at candidate loci were visually inspected using Artemis, which was also used to capture images for figures.^29^

### Validation experiments

A total of 18 mutants were selected from the KEIO library to validate predictions about susceptibility to fosfomycin made by TraDIS-*Xpress*.^30^ These included genes in the phosphonate uptake and metabolism and phosphorus import systems identified as major contributors to fosfomycin susceptibility as well as a set of randomly selected control genes not expected to have any impact on fosfomycin susceptibility. Both replicate mutants present in the KEIO collection (the collection contains two independent insertion mutants for each gene in BW25113) were analysed. Mutants were tested for their susceptibility to fosfomycin by determination of fosfomycin MIC. All experiments were duplicated (giving at least four datasets for each gene: two repeats from each of the two mutant alleles of each gene). BW25113 was included in all experiments as a control.

The potential for the bis-phosphonate etidronate to antagonize fosfomycin was evaluated using chequerboard assays where dilutions of fosfomycin (from 16 to 0.125 mg/L) and dilutions of etidronate (from 400 to 0.4 mg/L) were combined. Plates were incubated for 24 h at 37°C in a FLUOstar Omega plate reader (BMG Labtech) with OD measurements being taken every 15 min to capture growth kinetics.

## Results

### Susceptibility of BW25113 to fosfomycin

The MIC of fosfomycin for BW25113 was determined to be 4 mg/L in both MH and LB broth, and cultures were prepared in LB broth without or with fosfomycin at 0.25×, 0.5×, 1× and 2× MIC, combined with IPTG at two concentrations to induce transcription from the outward-transcribing transposon promoter, or without IPTG to allow promoter repression. Each condition was performed in duplicate to give a total of 30 independent experiments, and each was inoculated with ∼10^7^ cfu from the transposon mutant library. After incubation overnight, DNA was extracted from all cultures, and transposon insertion sites were identified using TraDIS-*Xpress*. Figure S2 shows the concordance between independent repeats and the impacts of increasing drug concentrations on numbers of nucleotide sequence reads locating to each gene of the whole genome.

### Genes involved in fosfomycin susceptibility

**Figure 1.**
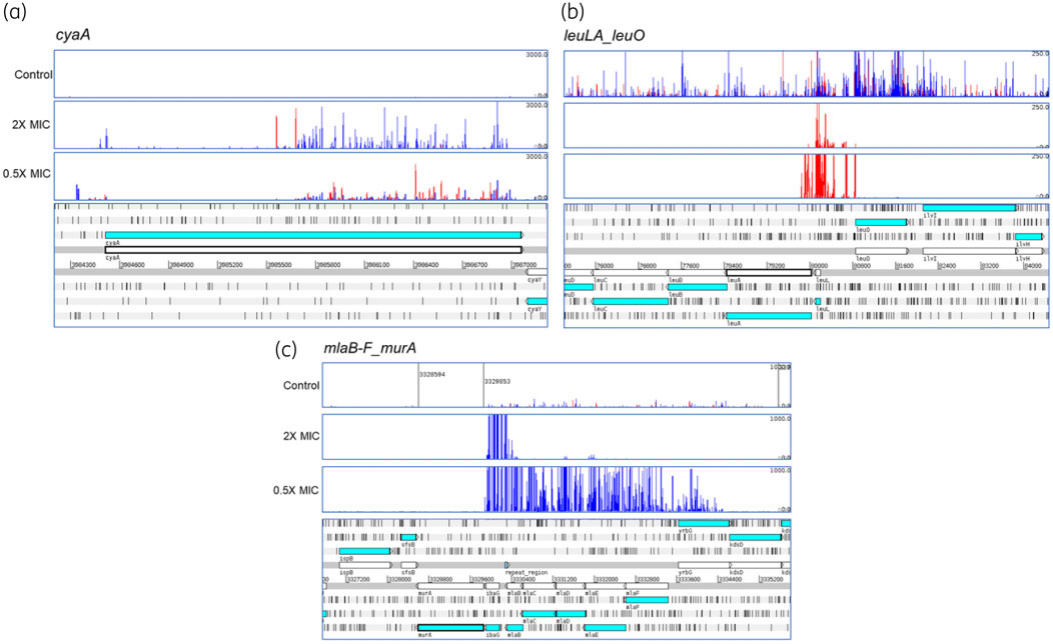
Differential selection of transposon mutants at *cyaA* (a), *leuO* (b) and *murA* (c). The bottom of each panel illustrates the genomic context and the panels above illustrate the mapped reads. Red bars indicate reads orientated left to right and blue bars the opposite. The height of each bar reflects the abundance of each insert. Data shown are from conditions with IPTG present as an inducer.

Whilst exposure to different concentrations of fosfomycin identified some concentration-dependent genes, 31 were involved in susceptibility under all fosfomycin conditions (Table 1, Figure S3). These included the majority of known, chromosomally encoded loci that determine fosfomycin susceptibility, with strong signals identified for *murA*, the target for fosfomycin, *glpT* and *cyaA*. This validates the specificity of TraDIS-*Xpress* in identifying genes involved in fosfomycin susceptibility. The TraDIS-*Xpress* method also proved able to assay essential genes. For example, *murA* is an essential gene but mutants with inserts that are positioned upstream and in the same orientation as *murA* were highly enriched in the presence of IPTG (Figure 1). These mutants will over-express *murA*, which will help the target saturate the activity of fosfomycin. The high density of the library also allows very high-resolution analysis; for example, *cyaA* was identified as a significant mechanism but enrichment of inserts was restricted to that part of the gene encoding the regulatory domain (Figure 1). Mutations within this domain (but not the remainder of the protein) have previously been reported as being important in determining fosfomycin susceptibility and TraDIS-*Xpress* identified that part of the gene encoding this domain. However, no signal was identified at the *uhpT* locus, which has also previously been implicated in fosfomycin import, although it is not expressed in the test conditions so knockout mutations would have had no selective advantage.

**Table 1.** Loci significantly altered in all exposure conditions

Locus	Predicted impacts of insertion^a^	Function and interpretation
*ahr*	Over-expression	Aldehyde reductase
*alsR*	Inactivation and up-regulation of *alsBACEK*	Allose binding transporter
*cmk*	Inactivated	Cytidylate kinase; nucleotide binding
*cra*	Inactivated	Catabolite repressor/activator. Global regulator of carbon metabolism
*crp*	Antisense knockdown	Global regulator and carbon catabolite repression
*cyaA*	Inactivation of regulatory domain	Adenylate cyclase; regulates cyclase activity by carbon source
*dacB*	Intragenic insert at one site	Peptidoglycan biosynthesis
*galU*	Inactivated	Synthesis of UDP-d-glucose
*glpE*	Inactivation and overexpression of *glpD*	Glycerol metabolism
*glpG*	Inactivated	Membrane protein protease
*glpK*	Inactivated	Glycerol kinase; key player in glucose control of glycerol metabolism
*glpT*	Inactivated	Glycerol uptake; known fosfomycin importer
*glpX*	Protected	Fructose bisphosphatase
*guaA*	Inactivation	GMP synthesis
*ibaG*	Inactivated, inserts upstream of *murA*	Increase in *murA* expression
*leuA*	Protected and over-expression of *leuO*	Regulation of stringent response, pleiotropic effects from *leuO*
*leuL*	Inactivated and over-expression of *leuO*	Pleiotropic effects from *leuO*
*mlaB-F*	Inactivated, inserts upstream of *murA*	Peptidoglycan recycling; fosfomycin target expression increased
*murA*	Upstream inserts	Peptidoglycan biosynthesis; fosfomycin target; expression increased
*mutL*	Protected	DNA repair
*nagB*	Inactivated with up-regulation of *nagE*	Over-expression of sugar importer (*nagE*)
*phnC-M*	Inactivated (all 14 members of operon in sense)	Phase-variable phosphonate transporter and degradation complex
*phoU*	Inactivation (downstream of *pstB*)	Repressor of *pstABCS* phosphate uptake system
*pstABCS*	Inactivated	Phosphate uptake system
*ptsH*	Inactivated	Decoration of imported sugars with phosphates
*purA*	Inactivated	Purine biosynthesis
*rfaH*	Inactivated (inserts antisense to *tatD*)	Transcription anti-terminator
*tatD*	Protected	DNA repair
*treC*	Inactivated	Hydrolyses trehalose to glucose
*waaFGP*	Inactivated	LPS branching
*yhfA*	Protected and up-regulated	Conserved hypothetical product, known to be Regulated by CRP

a Over-expression indicates selective advantage for insertions upstream. ‘Protected’ indicates a loss of insertion mutants in the selective condition.

### Identification of new mechanisms that reduce susceptibility to fosfomycin

As well as loci known to be involved in determining fosfomycin susceptibility, several new loci were identified by TraDIS-*Xpress*. These included the *cra*, *crp*, *cyaA*, *galU*, *glpK*, *glpX* and *treC* genes, which are involved in control of available glucose within the cell (Table 1) and will influence expression of the glucose importers that are known routes of entry for fosfomycin. Mutants that over-expressed *leuO*, a pleiotropic regulator with known roles in control of stress responses, were strongly selected by fosfomycin (Figure 1).

**Figure 2.**
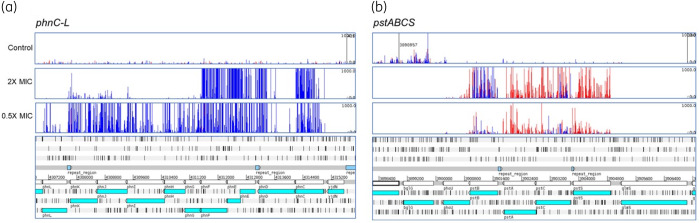
Inactivation of the *phn* (a) and *pst* (b) operons is selected by fosfomycin. The bottom of each panel illustrates the genomic context and the panels above illustrate the mapped reads. Red bars indicate reads orientated left to right and blue bars the opposite. The height of each bar reflects the abundance of each insert.

Mutants with transposon insertion mutations within the *phn* operon coding for phosphonate uptake and degradation were very strongly selected by fosfomycin (Figure 2). The phosphonate uptake and degradation operon comprises 14 genes (*phnCDEFGHIJKLMNOP*) coding for an ABC importer (PhnCDE), a regulator (PhnF), a multi-subunit methylphosphonate degradation complex (PhnGHIJKL), which includes the carbon–phosphorus bond cleavage activity (PhnGHIJ), and additional functions required to yield phosphate from phosphonate (PhnMNOP). The *phn* operon has been reported to be cryptic in *E. coli* K12 due to three, not two, copies of an 8 bp tandem repeat within *phnE* (encoding the integral membrane component of the importer) that cause a frameshift.^31^ Sequencing of the parent strain of the transposon library, and mapping of reads obtained from TraDIS experiments after fosfomycin exposure confirmed that the BW25113 parent strain, used to make the transposon mutant library, also has three copies of the tandem repeat within *phnE*, which should therefore not be expressed. However, following growth with fosfomycin at 2× MIC, transposon insertions in the *phnC*, *phnE* and *phnF* genes conferred a large selective advantage (Figure 2a). These insertions are all oriented such that the transposon promoter will transcribe the rest of the operon, most likely leading to its expression, and suggesting that the phosphonate degradation complex encoded by these genes can modify or degrade fosfomycin. At lower concentrations (0.5× MIC) of fosfomycin, insertion mutations in the same orientation were observed across most of the operon but not within *phnMNOP*, suggesting that at this lower concentration these gene products alone provide sufficient inactivation of fosfomycin to confer a significant selective advantage.

Insertion mutations in the *pstSACB* operon coding for an ABC phosphate importer also conferred a selective advantage during growth with fosfomycin (Figure 2). Insertion mutations in this operon generally showed an antisense orientation bias for at least two of the genes, which probably reflects the relative selective advantage or disadvantage of inactivation and altered expression of the operon components resulting from the different insertions sites of the transposon and its outward-directed promoter.

In contrast, the DNA repair functions encoded by *mutL* and *tatD* were beneficial for growth with fosfomycin, as insertion mutants for these genes were lost at all fosfomycin concentrations.

Changes in the expression of some bacterial cell functions reduce susceptibility to several antibiotics simultaneously. These functions include efflux transporters, porins and proteins that regulate their expression directly or indirectly, such as AcrR, MarA and SoxR.^32^ The TraDIS-*Xpress* data indicate that none of these functions contributes to susceptibility at any of the fosfomycin concentrations tested, suggesting that selection of cross-resistance to other antibacterials by fosfomycin is limited.

### Validation of targets

**Figure 3.**
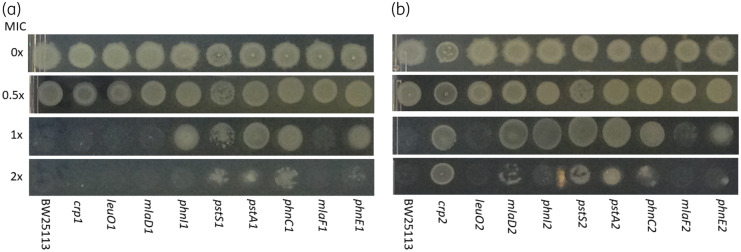
Validation of specific mutants by inoculation onto agar containing different fosfomycin concentrations. Panels (a) and (b) represent analysis of both independent mutants for each gene present in the KEIO collection. For each strain, 5 μL spots representing ∼10^4^ cfu were inoculated and incubated overnight at 37°C. Concentrations are indicated as fractions of the MIC (4 mg/L) for BW25113.

To test the predictions made by TraDIS-*Xpress* we tested nine pairs of insertion mutants from the KEIO collection for susceptibility to fosfomycin by growth on LB agar containing different concentrations of the drug (Figure 3). As expected, BW25113 was inhibited by the MIC. Growth of both the *leuO* mutants was also inhibited at the MIC, and this is also expected as the TraDIS-*Xpress* data predicted that altered *leuO* expression, rather than inactivation, was important for reduced fosfomycin susceptibility. For all the other mutant pairs, one or both showed a 2- to 4-fold increase in MIC of fosfomycin compared with the BW25113 parent strain. These mutant pairs included three for the *phn* operon and both the *pst* mutants tested (Figure 3). Given the indicated role for the phosphonate uptake system, we tested whether addition of an exogenous phosphonate would impact fosfomycin susceptibility and found that 100 mg/L of the bis-phosphonate etidronate increased the MIC of fosfomycin against BW25113 by 2- to 4-fold. In addition, growth curve experiments indicated that the addition of 100 mg/L etidronate resulted in improvements in growth rate of BW25113 cultures supplemented with different concentrations of fosfomycin (Figure S3). Etidronate alone showed no antibacterial activity (Figure S3).

## Discussion

The mechanisms of fosfomycin action and resistance known to date have largely been elucidated by the study of individual mutants and isolates that demonstrate fosfomycin resistance.^18^ Here, we used TraDIS-*Xpress* to assay the role of the whole genome of *E. coli* in fosfomycin susceptibility in a series of parallel experiments. The data showed the sensitivity of this approach, with many of the known chromosomal mechanisms of resistance being identified as important contributors to fosfomycin susceptibility (apart from *uhpT*, which is not expressed in the assay conditions used). Additionally, TraDIS-Xpress was also able to identify domains within targets that are important; for example, the regulatory domain of CyaA is known to be involved in fosfomycin susceptibility by controlling cAMP levels and therefore expression of *glpT* and *uhpT.*^19^ Inactivation of the CyaA domain responsible for this function results in lower expression of both GlpT and UhpT importers, and reduced fosfomycin susceptibility. TraDIS-*Xpress* clearly indicated the role for this regulatory domain but not the rest of *cyaA* (Figure 1). In line with the identification of *cyaA* as important, our data also identified inactivation of *glpT* as providing a selective advantage with fosfomycin as expected, although there was no signal for *uhpT*.

Traditional TraDIS experiments have not been able to assay many essential genes, which often reduce susceptibility to antibiotics. However, the current work demonstrates that TraDIS-*Xpress* did assay essential genes following growth in fosfomycin, as exemplified by the selection of many mutants with transposon insertions upstream of *murA* and oriented to promote transcription from the outward-transcribing transposon promoter. These insertions most likely increase expression, increasing the number of copies of MurA beyond that which can be inhibited by fosfomycin.

In addition to genes known to be involved in fosfomycin resistance, a wider set of new loci were also identified as contributing to susceptibility to the drug (Table 1). These included genes involved in glucose metabolism, which are likely to mediate expression of the glucose importers that facilitate transport of fosfomycin across the inner membrane. This extends our current understanding of how central metabolism can impact susceptibility to fosfomycin and shows how specific growth conditions and associated gene expression may influence susceptibility to this drug by altering gene regulation.

These experiments also found that knockout mutations in *mutL* and *tatD* were selectively disadvantageous for growth in fosfomycin. These genes encode DNA repair functions, suggesting that fosfomycin causes some DNA damage resulting from altered metabolism following inhibition of the primary target, MurA, and consequent bactericidal effect. This has been proposed as a common impact of many bactericidal drugs.^33–35^

The TraDIS-*Xpress* data indicated that transposon insertions into the phosphonate uptake and catabolism operon (*phnCDEFGHILJLMNO*) provided a considerable selective advantage for growth in fosfomycin. All these insertions were oriented such that the transposon outward-transcribing promoter would promote expression of downstream genes, suggesting that the products of this system can inactivate fosfomycin. At 0.5× MIC fosfomycin, insertions were found across most of the operon, but not within *phnMNOP*, suggesting that at lower fosfomycin concentrations one or more of these functions is sufficient for fosfomycin inactivation. At the highest fosfomycin concentration investigated, insertions were upstream of *phnG*, and orientated towards *phnG-P*, thereby over-expressing the lyase components. The lack of transposon insertions in the reverse orientation within *phnCDE* indicates the selective advantage is not likely to be due to inactivation of transport, which should remain inactive throughout the experiment due to the *phnE* frameshift mutation. There was, however, a phenotype for the *phnC* mutant, and *phnCD* will be expressed as they are upstream of the frameshift even though *phnE* is inactive. Therefore, these results indicate that it is most likely expression of the phosphonate metabolism system, *phnGHIJKLMNOP*, that provides a selective advantage during growth with fosfomycin.

Whilst UhpT and GlpT are known import systems for fosfomycin, mutation of either the PhnC-M or PstBCSA system was strongly selected by fosfomycin exposure (Figure 2).

The PstBCAS phosphate transporter was also identified as a likely mechanism of fosfomycin entry into the cell, as insertion mutations into the *pstBCAS* operon conferred a selective advantage during growth with fosfomycin (Figure 2). These data suggest that multiple importers, including novel systems, are involved in fosfomycin susceptibility. Testing of defined mutants in both the Phn and Pst systems confirmed a phenotype, with the mutants able to grow above the MIC of the drug (Figure 3).

In addition, growth in the presence of the bis-phosphonate etidronate resulted in limited but consistent (2-fold increase in the MIC) rescue of growth in the presence of fosfomycin. Figure S4 shows the impact of addition of 100 mg/L of etidronate on growth in the presence of a series of concentrations of fosfomycin, with improved growth evident at most concentrations (etidronate had no intrinsic antimicrobial activity). These data are supportive of specific phosphonate importers being important routes of entry into the cell for fosfomycin. The potential for phosphonate moieties to mediate import of molecules into the cell may have potential utility. A major challenge in therapy is getting drugs into cells and new routes to modify molecules and promote their uptake are likely to enhance efficacy of drugs. It has become clear in recent years that most drugs cross the inner membrane by active import rather than passive diffusion and identifying side groups that may improve uptake by changing importer specificity is important.^36^

**Figure 4.**
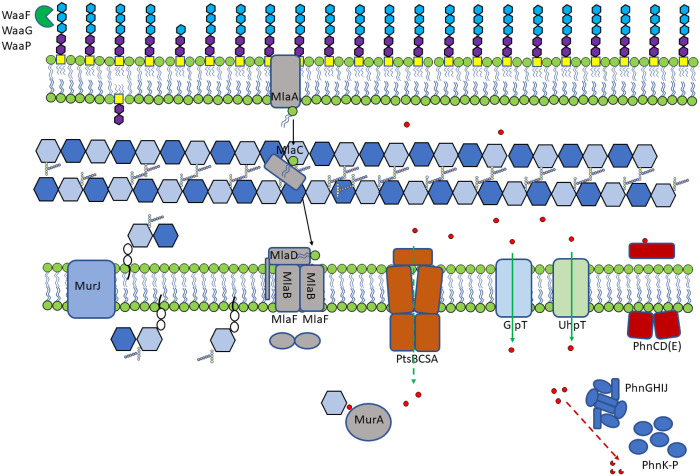
Summary of pathways implicated in fosfomycin entry into the cell. Proposed model of roles for genes identified as being involved in fosfomycin susceptibility. Fosfomycin is indicated by red circles and known and potentially novel mechanisms of uptake are indicated by solid and dashed green arrows, respectively.

One major worry with some drugs is selection of cross-resistance to other agents, often mediated by generic mechanisms of resistance, including multidrug efflux and/or porins, where expression changes can influence accumulation of many drugs.^32^ There was not a strong signal for these pathways after fosfomycin exposure, which suggests that the major mechanisms of fosfomycin resistance (we propose a model in Figure 4) are likely to be relatively specific and not influence other classes of agent. This is potentially important as, whilst fosfomycin resistance is not hard to select, a key feature of this drug is its activity against strains resistant to other drugs, in particular various β-lactams.

Taken together, these data show a genome-wide analysis of genes not known previously to be involved in susceptibility to fosfomycin. These include a role for Pst as a transporter and the phosphonate catabolism pathway as a putative inactivator of fosfomycin.

## Supplementary Material

dkaa296_supplementary_data
