# Supplementary material for: A genome-wide analysis of Escherichia coli responses to fosfomycin using TraDIS-Xpress reveals novel roles for phosphonate degradation and phosphate transport systems
Source: J Antimicrob Chemother. 2020 Aug 5;75(11):3144–51. doi: 10.1093/jac/dkaa296 (PMC7566553; doi:10.1093/jac/dkaa296)
Supplement: dkaa296_supplementary_data [file dkaa296_supplementary_data.zip › 20-0080-Supplementary data.docx]

**Supplementary data**

**Supplementary figures**

**Figure S1.** Overview of experimental conditions and replicates

**
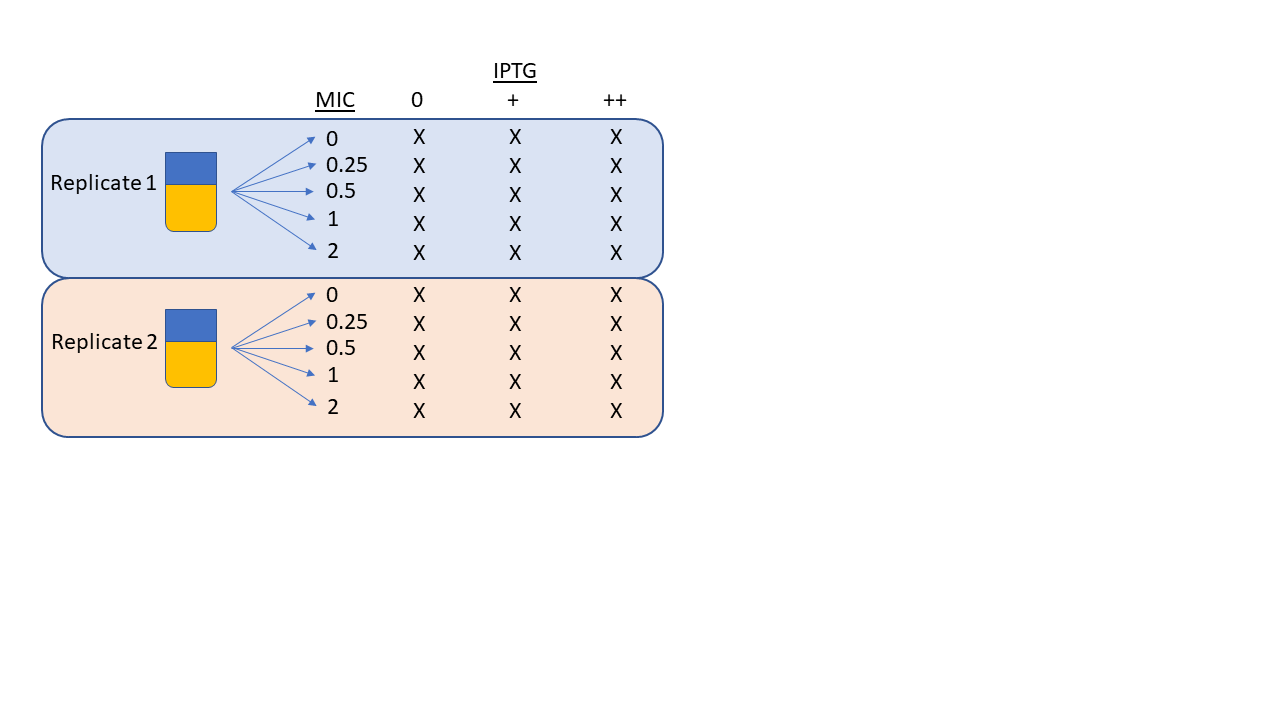
**

**Figure S1.** Overview of experimental conditions and replicates**.** A total of 30 experiments were completed (marked by ‘X’s)

**Figure S2. Comparison of reads per gene under all conditions**

**
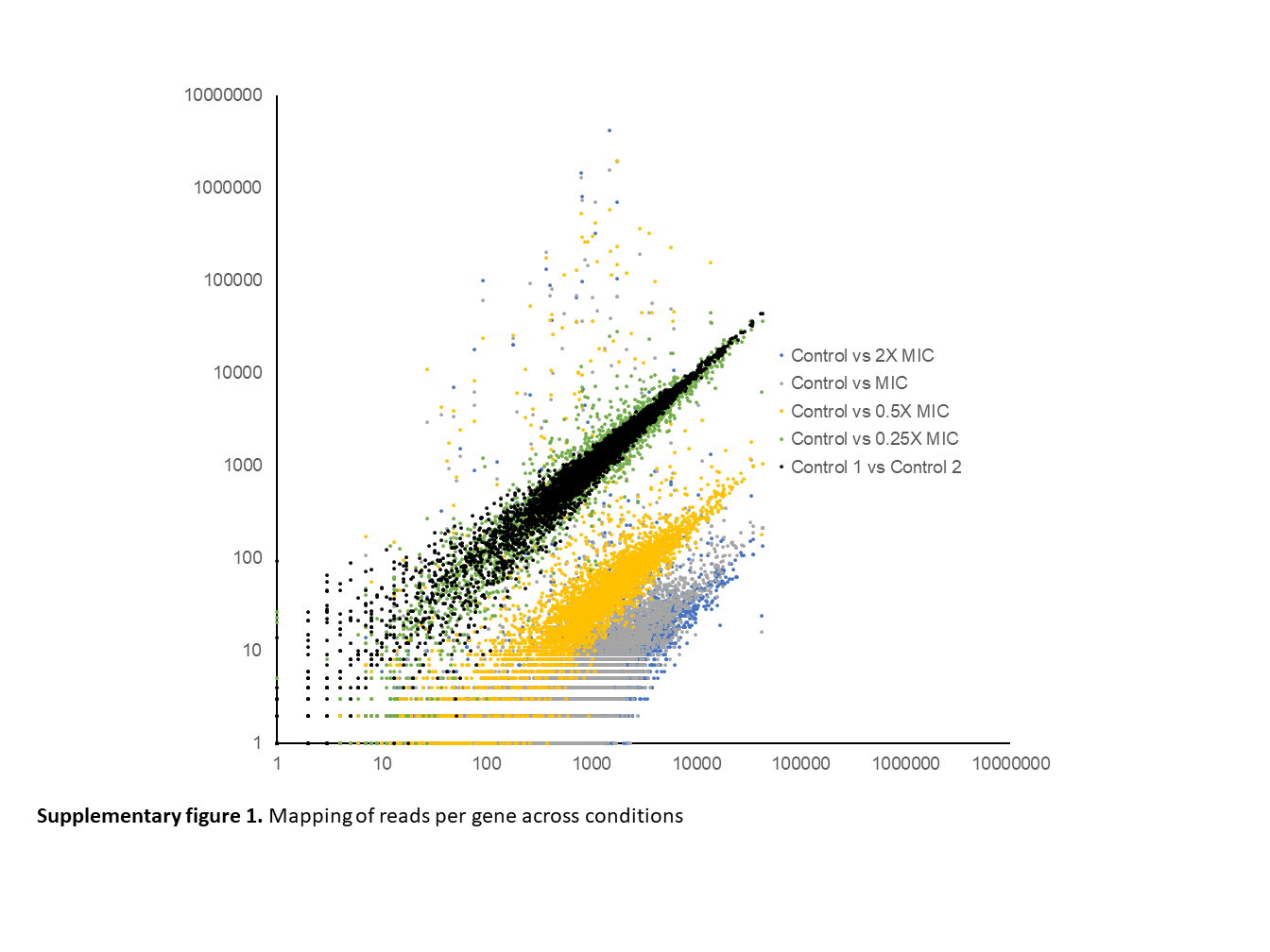
**

**Figure S2.** Reads mapped against each gene are plotted. The black dots show the concordance between control replicates which are mapped against each other. Coloured dots show the average number of reads mapped in the controls (y-axis) against the average number for each drug exposure (x-axis). Increasing concentrations of fosfomycin select for greater numbers of fewer mutants, hence the number of reads associated with a few genes increases greatly at the expense of most genes, for which the number of reads goes down .

**Figure S3. Heatmap comparing genes selected by fosfomycin at different exposure conditions**

**
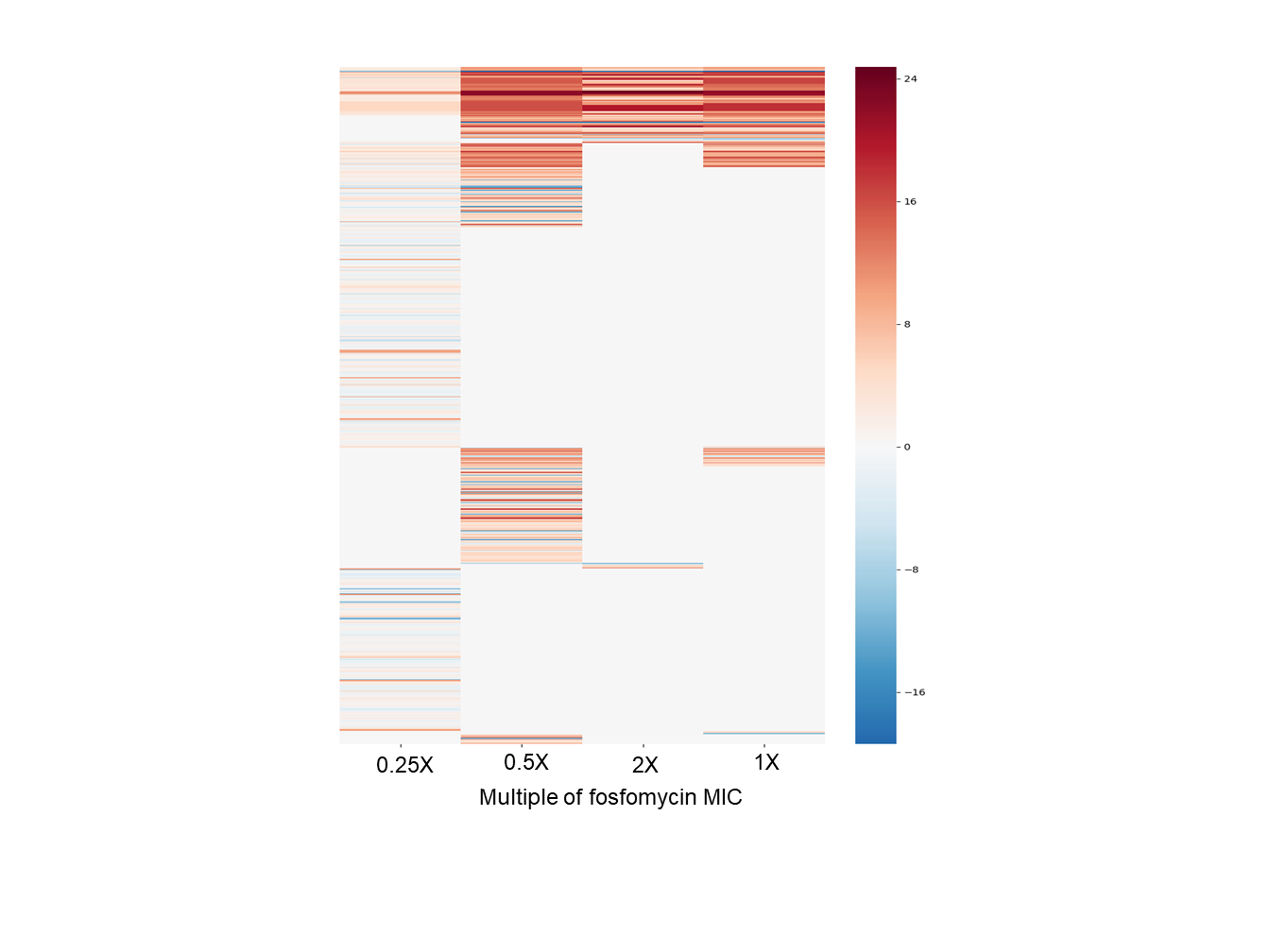
**

**Figure S3.** The heatmap illustrates similarilties in insert patterns identified under the different drug exposure conditions. A core set of genes are clearly seen across all conditions.

**Figure S4**. The impact of etidronate on fosfomycin susceptibility


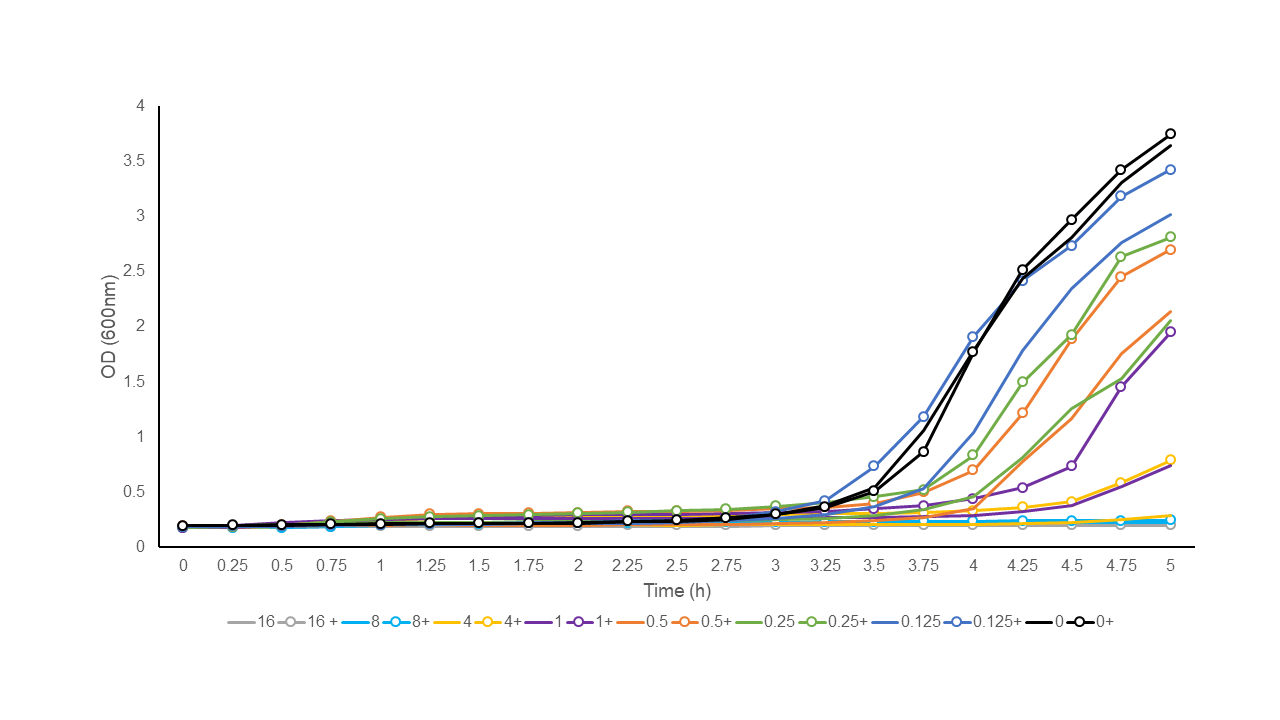


**Figure S4**. Growth of BW25113 in the presence of different concentrations of fosfomycin (indicated by different colours), each in the presence (open circles) or absence (plain lines) of etidronate (100 mg/L).
